# Establishing a Reproducible Murine Animal Model of Single Anastomosis Duodenoileal Bypass with Sleeve Gastrectomy (SADl-S)

**DOI:** 10.1007/s11695-018-3254-4

**Published:** 2018-04-25

**Authors:** Montana Laura, Lamon Mylene, Barrat Christophe, Hansel Boris, Magnan Christophe, Arapis Konstantinos

**Affiliations:** 1 Department of Digestive and Metabolic Surgery, Centre Intégré Nord Francilien de l’Obésité, University Hospital Avicenne, 125 rue de Stalingrad, Bobigny, 93000 Paris, France; 2 Team of Regulation of Glucose Homeostasis by Nervous System (REGLYS), University of Paris (7) Diderot-CNRS UMR8251, Bâtiment Buffon, 3ème étage, pièce 340A, case courrier 7126, 4 rue Marie Andrée Lagroua Weill-Halle, 75205 Paris Cedex 13, France; 3 Department of Diabetes and Nutrition, Bichat-Claude Bernard University Hospital, 46, rue Henri Huchard, 75877 Cedex 18 Paris, France; 4 Department of General and Digestive Surgery, University Hospital Bichat-Claude Bernard, 46, rue Henri Huchard, 75877 Cedex 18 Paris, France

**Keywords:** Animal models, Metabolic surgery, SADI-S, Duodenal switch, Biliopancreatic diversion

## Abstract

Single-anastomosis duodenoileal bypass with sleeve gastrectomy (SADI-S) is a simplified biliopancreatic diversion. The objective of this study was to develop a reproducible animal model for SADI-S. We used three techniques for duodenal exclusion and duodenoileal anastomosis: (a) surgical clip and side-to-side anastomosis, (b) ligation and side-to-side anastomosis and (c) sectioning the duodenum, closing the duodenal stump and end-to-side anastomosis. We recorded the surgical technique and complications for each method. Twenty-five of 31 rats survived to the end of the study period. One death occurred from accidental anaesthesia overdose and the others from anastomosis leak. Four duodenal exclusions had repermeabilised at necropsy. Our murine model of SADI-S can be consistently reproduced. Sectioning the duodenum is preferable to avoid repermeabilisation of the duodenum.

## Introduction

Single-anastomosis duodenoileal bypass with sleeve gastrectomy (SADI-S) was proposed by Sànchez-Pernaute A. et al. [1] as a modification of the original biliopancreatic diversion with duodenal switch. The advantages of SADI-S include the single-anastomosis procedure, no mesenteric opening [2], pyloric preservation [3] and the physiological advantages of the “short circuit” for nutrient delivery from the stomach to the ileum [4]. Recently, the same team proposed this procedure as a second surgical option after sleeve gastrectomy with insufficient weight loss or weight regain [5]. Until our study, there was no animal model to investigate this procedure; we present a reproducible animal model for SADI-S.

## Methods

Thirty-one adult male Winstar rats (12–16 weeks old) were included in this study. All animals were housed in individual cages under constant ambient temperature (23 °C) and humidity, and were maintained in a 12-h light/dark cycle room.

### Preoperative Care

The night before surgery, the rats were kept on raised wire platforms to avoid coprophagy and fasted for approximately 12 h; only water was allowed ad libitum. An isoflurane anaesthetic chamber was used for anaesthesia induction, which was changed to nose cone flow for maintenance of anaesthesia during the procedure. Enrofloxacin 2.5%, 25 mg/kg, was administered intramuscularly for antibacterial prophylaxis, and 20 mL of normal saline solution was given subcutaneously for hydration (10 mL before surgery and 10 mL postoperatively).

### Surgical Procedure

Following induction, the abdomen was shaved with electric hair clipper from sternum to groin, and the rat was placed on a heating pad in dorsal recumbency. The skin was disinfected using an aqueous iodophor solution, and after identifying the xiphoid process, a 5-cm midline abdominal incision was made using a #11 surgical scalpel. After identifying the *linea alba*, a midline incision was made in the abdominal wall using an electrical scalpel. The abdominal cavity was then exposed using a surgical retraction system (Canica Rat Surgical Table, Ketchum Manufacturing Inc., Ottawa, Canada).

**Fig. 1:**
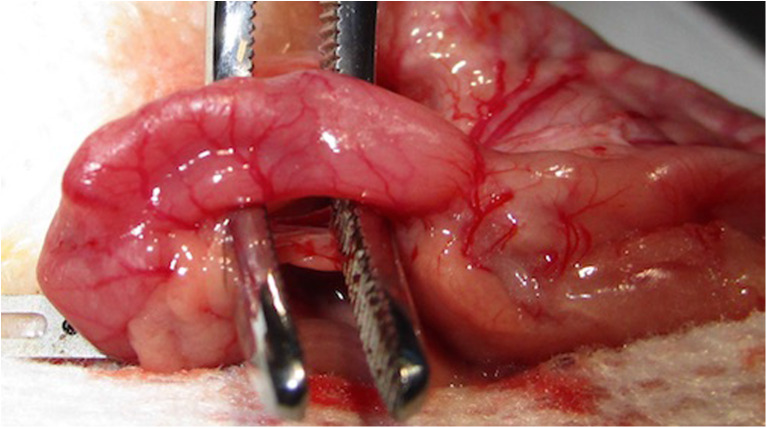
Duodenal dissection

**Fig. 2:**
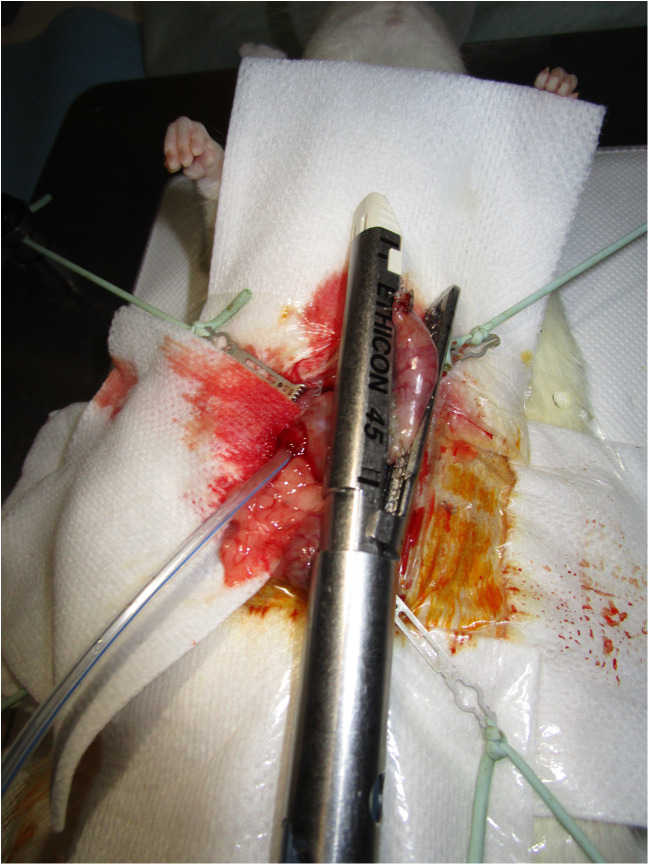
Gastric tube calibration through the duodenotomy

We performed a complete gastrectomy of the greater curvature using surgical scissors. The gastrosplenic ligament was sectioned after ligation with 4.0 absorbable sutures (4.0 Vicryl™, Ethicon Inc., Somerville, NJ). We continue with dissection of the duodenum (Fig. 1). Next, we passed an 8-FR catheter from the mouth to the duodenum or from the lateral duodenotomy (Fig. 2). A standard sleeve gastrectomy around the 8-FR catheter was performed using a blue reloadable laparoscopic stapler (Echelon™ Flex™, Ethicon). We then externalised and measured the length of the small intestine from the ileocolic junction for approximately 30% of the total length of the intestinal tract; approximately 35 cm. This point is the location of the future duodenoileal anastomosis.

Three methods were used for duodenal exclusion and duodenoileal anastomosis:

- Ligation of the duodenum 5 mm from the pylorus using 3.0 nonabsorbable suture (Mersutures™, Ethicon) followed by side-to-side duodenoileal anastomosis using a continuous pattern with absorbable 6.0 suture (PDS™ II, Ethicon).
- Ligation of the duodenum 5 mm from the pylorus using a nonabsorbable surgical clip (Ligaclip MCM 20™, Ethicon) followed by side-to-side duodenoileal anastomosis using a continuous pattern with absorbable 6.0 suture (PDS™ II, Ethicon).
- Sectioning the duodenum 5 mm from the pylorus and closure of the duodenal stump in a continuous pattern with nonabsorbable 6.0 suture (Prolene™, Ethicon) followed by end-to-side duodenoileal anastomosis in a continuous pattern with absorbable 6.0 suture (PDS™ II, Ethicon). It is interesting to note that duodenal sectioning can be an alternative method of gastric tube calibration.

**Fig. 3:**
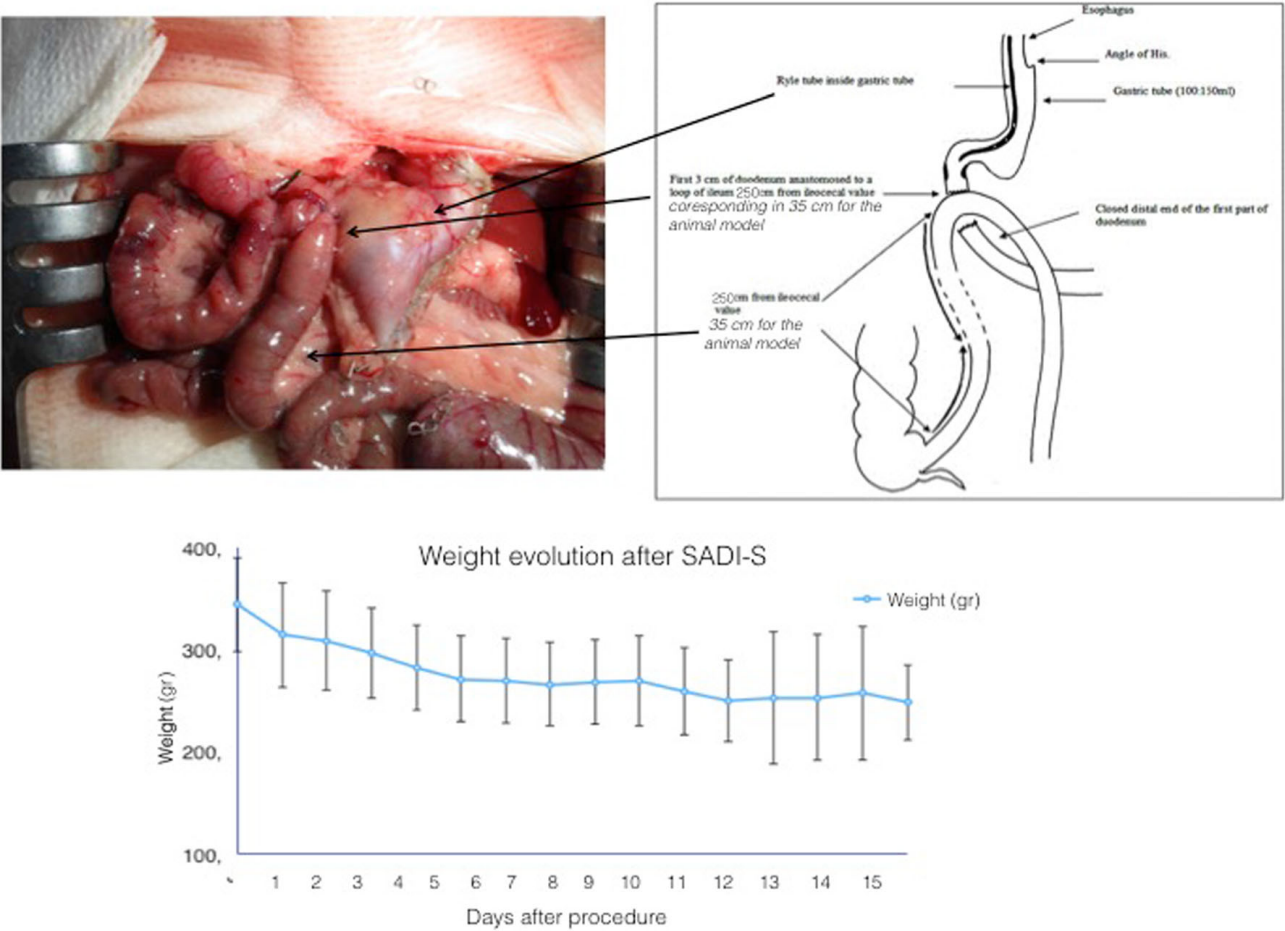
Final reconstruction with human equivalent, weight postoperative evolution

Following the anastomosis (Fig. 3), we returned the intestine to the abdominal cavity and closed the abdominal muscle using continuous 3.0 nonabsorbable sutures (Mersutures™, Ethicon). The abdominal skin was closed using an interrupted pattern with nonabsorbable 4.0 suture (Mersutures™, Ethicon).

### Postoperative Care

Immediately postoperatively, each rat recovered under a heat lamp with continuous monitoring until regaining consciousness. After recovery, the animals were transferred to a cage in isolation, kept on a raised wire platform, and fasted for the first 24 h. Liquid diet (Altromin C0200, Altromin Spezialfutter GmbH & Co. KG, Lage, Germany) was provided for the next 3 days. Antibiotics (enrofloxacin) were administered for 72 h. Full solid diet was administered on day 7 with a transition time of 3 days during which we provided liquid and solid diet simultaneously. At the end of the study period, the rats were sacrificed by decapitation after exploratory laparotomy under isoflurane anaesthesia. The brain was removed for immunohistochemistry studies measuring neuronal activity (quantification of cfos which is a marker of activated neurons).

### Data Collection

Operative time and anaesthetic death were recorded, and the study period, as our goal was to validate the technique in rats, was 2 weeks in duration. Body weight was recorded before surgery and every postoperative day, with each death investigated to identify complications. After 2 weeks, an exploratory laparotomy was performed to identify fistula, intestinal occlusion, or repermeabilisation of the duodenal exclusion. Data analyses are expressed as the mean ± standard deviation.

## Results

The mean operative time was 40 ± 15 min and we found no significant difference between the three procedures. The mean initial weight of the rats was 344 ± 34 g (range 263–475 g), and the mean postoperative weight was 261 ± 42 g (range 195–391 g) (*p <* 0,001) (Fig. 3). Six deaths occurred with one secondary to anaesthesia overdose. The other five deaths were caused by anastomosis leak between the first and the seventh postoperative day; three anastomotic leaks occurred in the side-to-side anastomosis group and two in the end-to-side anastomosis group.

During the exploratory laparotomy at 2 weeks, we identified two perianastomotic abscesses (1 in the side-to-side group and 1 in the end-to-side anastomosis group), and 4 cases of repermeabilisation of the duodenal exclusion (three cases with nonabsorbable suture and 1 case when a surgical clip was used).

## Conclusion

To our knowledge, ours is the first study evaluating the feasibility of an animal model for SADI-S. We chose this strain of Wistar rats because it is the strain most used in preclinical studies focused on obesity and diabetes. This strain is also highly sensitive to weight gain on hyperlipidic diet and quickly becomes insulin resistance. We experienced low overall mortality (5/31, 16.3%) and seven anastomotic leaks: four in the side-to-side anastomosis group and three in the end-to-side anastomosis group (*p* = 0.34). To decrease duodenal stump complications, we performed duodenal exclusion with either suture ligation or surgical clip ligation. However, four cases of repermeabilisation of the duodenal exclusion were seen, and these occurred with duodenal exclusion without sectioning the duodenum. In contrast, we encountered no duodenal stump complications when we sectioned the duodenum.

To create a larger gastric tube, we used an 8-FR calibration catheter (Fig. 2), contrary to the 7-Fr gauge that is usually proposed [6]. This difference may allow researchers to reproduce the difference between a standard sleeve gastrectomy calibration (38-FR) and calibration for SADI-S (54 FR) [1].

In the original technique described by Sanchez-Pernaute [1], a 200-cm common channel was used, and this was later changed to a 250-cm common channel to avoid malnutrition [7]. A 250-cm limb is slightly more than 1/3 of the entire bowel length if 6 m is considered the total length of the human bowel. In the rat, bowel total length is 80 cm to 1 m; therefore, we used a 35-cm common channel (Fig. 3). We observed that this length corresponded to a fourth vascular arcade from the ileocolic junction. We saw no cases of diarrhoea cases during the postoperative period.

In conclusion, this study represents the first report of a preclinical model for SADI-S. This technique will allow researchers to evaluate postoperative metabolic homeostasis and compare their findings with other standard surgical bariatric techniques.
